# Curative-Intent Cryoablation for Bone Metastases: Study Protocol for BOREALIS, a Prospective Multicentre Observational Investigation

**DOI:** 10.1007/s00270-026-04550-z

**Published:** 2026-08-19

**Authors:** Roberto Luigi Cazzato, Xavier Buy, Fernando Gomez, Frederic Deschamps, Dirk Arnold, Dimitrios Filippiadis, Jack Jennings, Niklaus Schaefer, Olivier Schneegans, Georgia Tsoumakidou, Bleranda Zeka, Claire Poulet, Nathalie Kaufmann, Jean Palussiere, Afshin Gangi

**Affiliations:** 1 Department of Interventional Radiology, University Hospital of Strasbourg, 1 place de l’hôpital, 67000 Strasbourg, France; 2 Department of Interventional Radiology, Institut Bergonié, Bordeaux, France; 3 Hospital Universitario y Politécnico La Fe, Valencia, Spain; 4 Departement of Interventional Radiology, Gustave Roussy, 94805 Villejuif, France; 5 Asklepios Tumorzentrum, Hamburg, Germany; 6 2nd Department of Radiology, University General Hospital “ATTIKON”, Medical School, National and Kapodistrian University of Athens, Athens, Greece; 7 Washington University, Saint-Louis, USA; 8 Departement of Nuclear Medicine, University Hospital of Lausanne, Rue du Bugnon 46, 1011 Lausanne, Switzerland; 9 Service de Médecine Nucléaire, Institut Public de Cancérologie Strauss Europe 17, Rue Albert Calmette, BP 23 Zéro 25, 67033 Strasbourg, France; 10 Diagnostic and Interventional Radiology Department, University Hospital of Lausanne, Rue du Bugnon 46, 1011 Lausanne, Switzerland; 11 Next Research, Cardiovascular and Interventional Radiological Society of Europe, Neutorgasse 9, 1010 Vienna, Austria; 12 School of Biomedical Engineering and Imaging Sciences, King’s College London, London, UK

**Keywords:** Cryoablation, Bone metastases, Response criteria, Oligometastatic disease, Curative-intent, Prospective oberservational study

## Abstract

**Purpose:**

Cryoablation (CA) is a well-established, safe and effective palliative treatment for painful bone metastases (BM). Data exploring its curative potential (i.e. radical clearing of the target lesion) is however very scarce, mostly coming from small retrospective studies. The BOne metastases REgistry for patients undergoing cryoAbLation with curatIve purpoSe (BOREALIS) aims to address this shortcoming by collecting prospective, multicentric real-world data on the outcomes of curative-intent CA for bone metastases.

**Methods:**

BOREALIS is an observational cohort study designed to follow for 1 year up to 50 participants with BM (< 5 cm) from oligometastatic or bone oligo-progressive disease treated with curative-intent CA. The primary outcome measure is the 12-months local progression-free survival rate, assessed per lesion using the newly developed BOREALIS tumour response criteria. Secondary outcome measures include complete response rate, fracture-free, progression-free, and overall survival; safety; patient-reported pain; and health-related quality of life (HRQoL). The study will also evaluate the feasibility of a comprehensive procedure integrating, when indicated, biopsy and bone consolidation within the same CA session. Results will be reported using mainly descriptive statistics. Patient enrolment opened in April 2025.

**Conclusion:**

BOREALIS intends to provide insights into the effectiveness of curative-intent CA for BM to inform the development of future clinical guidelines. BOREALIS is registered on ClinicalTrial.gov (NCT06480955).

**Supplementary Information:**

The online version contains supplementary material available at https://doi.org/10.1007/s00270-026-04550-z.

## Introduction

As systemic therapies continue to extend survival, an increasing number of patients present with oligometastatic (≤ 5 lesions) or oligoprogressive bone disease, highlighting the growing need for local treatment strategies delivered with curative intent at the treated site. In this context, cryoablation (CA) has been applied selectively [1]; however, high-quality prospective data evaluating its locally curative potential remain limited. Existing studies are constrained by small sample sizes, retrospective designs, and substantial heterogeneity in patient selection, treatment protocols, and follow-up methods [2–8]. This lack of robust prospective evidence limits the ability to accurately assess the oncologic performance of CA and prevents the establishment of standardized indications for its use with a locally curative intent for bone metastases (BM).

The BOREALIS registry seeks to address this evidence gap by generating rigorous multicentre prospective data on patients undergoing CA for oligometastatic or oligoprogressive bone disease. Its primary objective is to evaluate the 1-year local non-progressive disease rate, providing a clinically meaningful measure of local tumor control.

## Study Design and Site Selection

BOREALIS is an investigator-initiated, prospective, multicentre, single-cohort observational study. It is sponsored by the Cardiovascular and Interventional Radiological Society of Europe (CIRSE) and overseen by a scientific Steering Committee composed of experts in interventional radiology, oncology, and nuclear medicine, with extensive experience in the management of BM. The study is conducted in compliance with the Declaration of Helsinki, Good Clinical Practice, and all relevant European and national regulations and has received all necessary approvals from national or local ethics committees and, where applicable, regulatory authorities.

Sites in Europe and North America with long-standing (> 10 years) experience in CA of BM and a minimum annual volume of 20 bone CA procedures during the preceding 2 years were invited to participate, with seven sites across five countries expected to contribute. Patient enrolment began in April 2025.

## Study Population

Patients with BM from oligometastatic or bone oligo-progressive disease from solid tumours scheduled to receive curative-intent CA per standard-of-care shall be recruited upon giving informed consent. Eligibility criteria are listed in Table 1. Principal Investigators (PIs) are instructed to offer participation in BOREALIS to all eligible patients and to document any refusals or reasons for non-eligibility as appropriate.

**Table 1:** Patient inclusion and exclusion criteria

Inclusion Criteria
1. Bone metastases from oligometastatic disease (< 5 bone/visceral metastases) or bone oligo-progressive disease (visceral or osseous multi-metastatic disease stable under systemic treatment except 1–3 progressing bone metastases) from solid tumours
2. Number of target bone metastases ≤ 3
3. Size of target bone metastases < 5 cm (largest diameter)
4. Referral to local ablative treatment by multidisciplinary tumour board (MDTB)
5. Treatment by percutaneous cryoablation with curative intent (i.e., complete target bone metastases destruction) assessed as technically feasible by the IR in charge
6. Procedure performed with a cryoablation system from Boston Scientific

Exclusion Criteria
1. < 18 years old
2. Incapacity or refusal to give informed consent
3. Ongoing pregnancy
4. Health status not compatible with the minimum meaningful follow-up period (i.e., 12 months): − Karnofsky Performance Scale < 60%, or − Bone metastases originating from rapidly evolving tumour histologies not allowing reasonable life expectancy of at least 12 months, or − Life expectancy < 12 months
5. Infection of treatment site or systemic infection
6. Uncorrectable coagulopathy
7. Haematological disease (including multiple myeloma and plasmacytoma)
8. Concomitant radiation therapy or radiation therapy within 12 weeks before the planned cryoablation procedure

## Data Collection and Quality Control

Baseline data, obtained within 6 weeks before the planned CA procedure, includes demographics (age and sex), performance status (ECOG scale) and relevant medical history (primary origin, date of diagnosis and TNM classification, number and location of extra-osseus metastases, and details on prior or concomitant systemic bone-protective or anti-cancer treatments). Information specific to the target BM is also captured: date of diagnosis, location, radiographic feature (lytic, sclerotic, mixed, CT-occult), and history of prior local treatments. Upon completion of the planned CA session, procedural details, including information on any adjunctive biopsy or bone consolidation, are recorded for each target BM, together with the corresponding DICOM files. Follow-up data is collected at 1, 3, 6 and 12 months. Safety data is recorded from the CA procedure throughout the study, and PROMs are planned to be captured at each follow-up time point.

All data and images are collected via an electronic data capture (EDC) system (REDCap Cloud, nPhase, Inc, Version 1.7.3) through electronic case report forms (eCRFs) which incorporate pre-defined data-type constraints, range checks, and automated alerts to minimize missing or inconsistent entries and ensure data completeness and uniformity. A regular remote monitoring scheme is implemented for each participating site to verify data quality, identify potential issues related to patient enrolment and data collection, and ensure compliance with the study protocol and applicable guidelines and regulations. Source data verification may be performed as needed.

## Outcome Measures

A list of all outcome measures is summarized in Table 2. Response to treatment will be assessed by blinded central image review (BCIR) of standard-of-care CE-MRI, CE-CT or PET-CT scans. Unless indicated otherwise, all time-to-event outcome measures will be evaluated from the time of the first CA session.

**Table 2:** Primary and secondary outcome measures in BOREALIS

	Outcome measure	When	Reported per	Reported by
Effectiveness - Primary	Local progression-free survival rate	12 months	Lesion	BCIR
Effectiveness - Secondary	Complete local tumour response	12 months	Lesion	BCIR
	Duration of response (PR or CR)	Time-to-event	Lesion	BCIR
	Fracture-free survival	Time-to-event	Lesion	Investigator/BCIR
	Fracture-free survival rate	12 months	Lesion	Investigator/BCIR
	Target bone metastasis progression-free survival	Time-to-event	Patient	BCIR
	Bone-disease progression-free survival	Time-to-event	Patient	Investigator
	Extraosseous disease progression-free survival	Time-to-event	Patient	Investigator
	Overall progression-free survival	Time-to-event	Patient	Investigator
	Overall survival	Time-to-event	Patient	Investigator
	Systemic therapy-free survival	Time-to-event	Patient	Investigator
Feasibility	Technical feasibility	Treatment day	Lesion	Investigator
	Feasibility of patient management	Until end of study	Patient	Investigator
Safety	Incidence and severity of PCs and major AEs	Until end of study	Patient	Investigator
PROMs	Brief Pain Inventory – Short form	1, 3, 6 and 12 months	Patient	Patient
	EORTC QLQ-C30 questionnaire	1, 3, 6 and 12 months	Patient	Patient

PCs: procedural complications, AEs: adverse events, PROMs: patient-reported outcome measures, EORTC: European Organisation for Research and Treatment of Cancer, BCIR: blinded central image review. Duration of response will be measured from the time of first documented PR or CR. All other time-to-event outcome measures will be assessed from the time of the first CA session

### BOREALIS Tumour Response Criteria

Evaluating local treatment response in BM remains challenging due to limitations of existing response criteria. Size-based approaches, such as RECIST [9], are inadequate for bone lesions where size is not always a reliable indicator of tumour viability and treatment response is more often reflected by structural, density, or metabolic changes. Structural or qualitative criteria, such as the MD Anderson criteria [10], lack clear quantitative thresholds, resulting in inter-observer variability and limited reproducibility. While criteria based on tumour viability or metabolic activity are central to response assessment, they often fail to capture relevant morphologic changes such as clinically significant increases in lesion volume [11–13]. Overall, these limitations highlight the need for an updated assessment framework that reflects imaging modalities currently used in clinical practice and integrates complementary morphologic and viability-related features with standardized, reproducible thresholds.

**Fig. 1:**
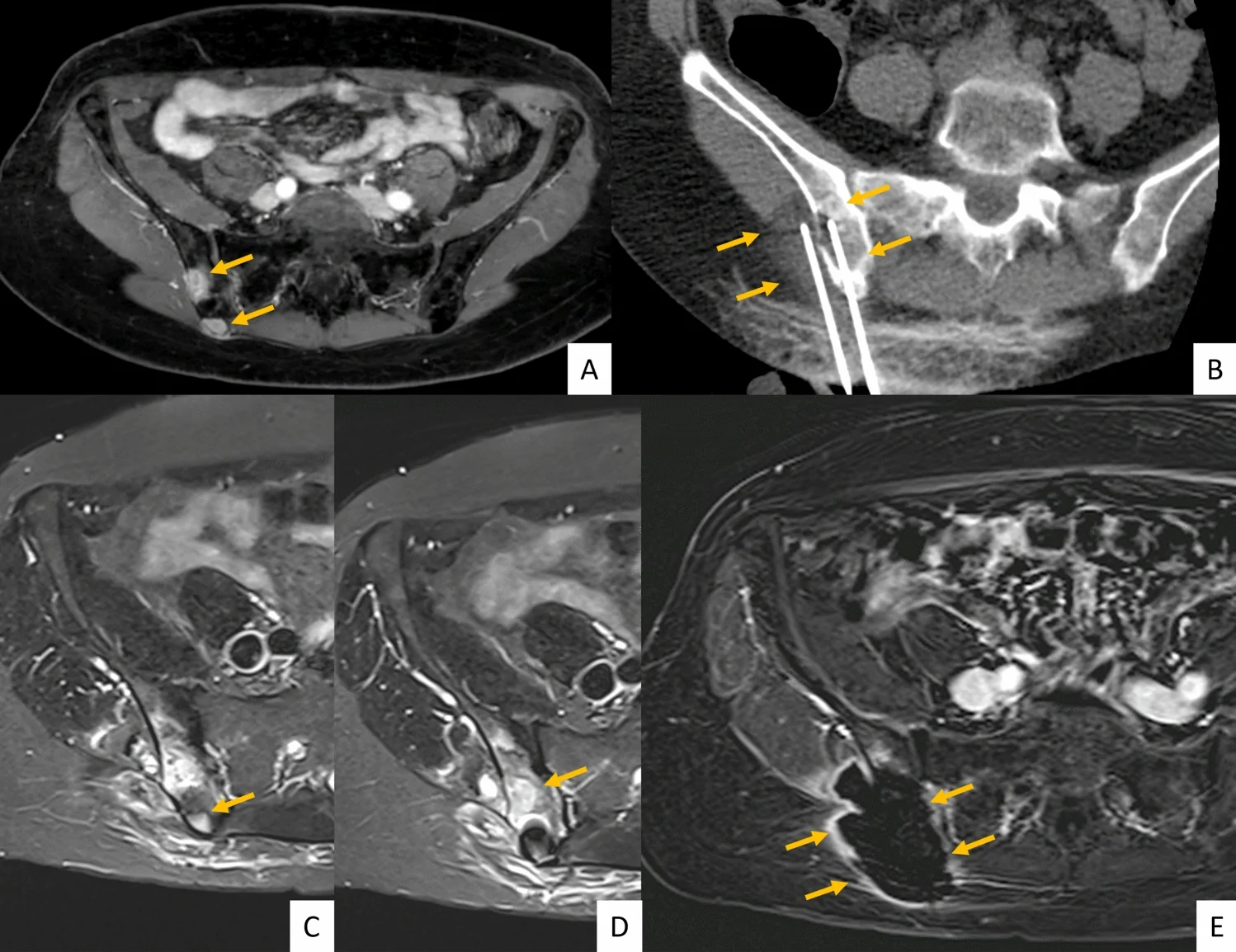
**Example of complete response (CR) assessed by CE-MRI**. 55-year-old female patient with two single, biopsy-proven, bone metastases of breast cancer in the right iliac bone.** A** Axial contrast-enhanced T1-weighted subtracted sequence showing the two metastases (arrows) respectively sized 1.6 and 1.2 cm (volumes: 1.9 and 0.5 cm^3^, respectively).** B** Given the oligo-metastatic scenario, the patient underwent percutaneous cryoablation with local curative intent; of notice the large iceball (arrows) encompassing the two metastases.** C**–**D** Axial T2-STIR sequences obtained at 1-month follow-up showing size stability (1.6 and 1.2 cm; volumes 1.9 and 0.5 cm^3^, respectively) for both metastases (arrows); and **E** axial contrast-enhanced T1-weighted subtracted sequence obtained at the same time interval, showing a large area of necrosis (arrows) encompassing the two metastases (i.e. no contrast-enhancement of the two target metastases), thus being consistent with a complete response (CR)

**Fig. 2:**
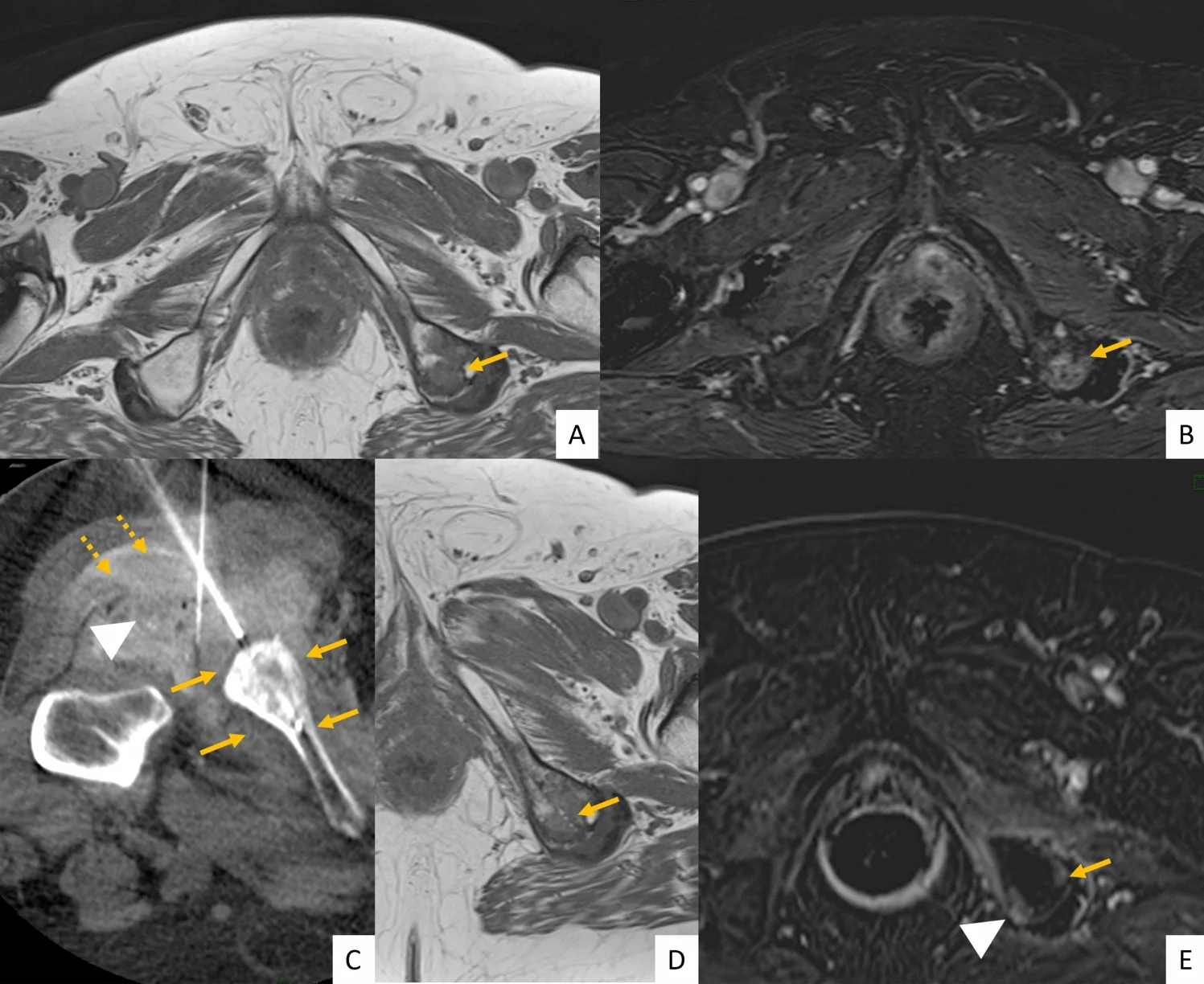
**Example of partial response (PR) assessed by CE-MRI**. 72-year-old male patient with a single biopsy-proven metastasis of prostate cancer in the left ischiatic bone. **A** Axial unenhanced T1 and **B** axial contrast-enhanced T1-weighted subtracted sequences showing the target metastasis (arrows) sized 1.9 cm (volume 4.5 cm^3^). **C** Given the oligo-metastatic scenario, the patient underwent percutaneous cryoablation with local «curative intent»; of notice the iceball (arrows) extending in the nearby soft-tissues; and the large hydrodissection (dotted arrows) to protect the sciatic nerve (arrowhead). **D** Axial T1-weighted sequence obtained at 1-month follow-up showing lesion stability in terms of size, and **E** axial contrast-enhanced T1-weighted subtracted sequence obtained at the same time interval showing a large area of necrosis (arrow) along with a persistent peripheral pseudo-nodular contrast-enhancement (sized 1.5 cm; volume 1.5 cm^3^, corresponding to a decrease of viable tumor volume larger than 30%) on the medial side of the ischiatic bone (arrow head), thus being consistent with a partial response (PR)

**Fig. 3:**
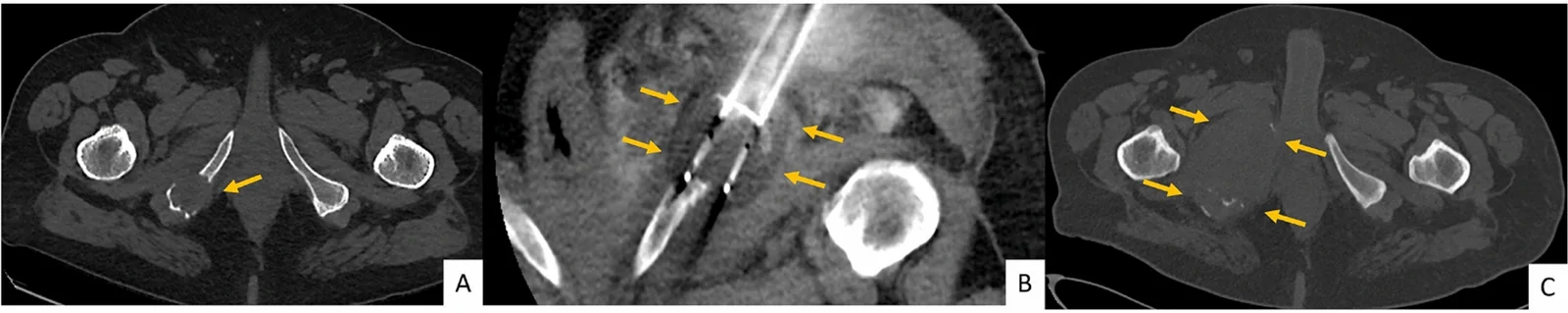
**Example of progressive disease (PD) assessed by CE-CT**. 53-year old male patient with a biopsy-proven metastasis of kidney cancer with sarcomatoid components (ISUP 4) of the right ischiatic bone, progressing despite systemic treatment. All the other known metastases were stable, thus being consistent with an oligoprogressive bone disease. **A** Axial computed tomography image showing the target metastasis (arrow), sized 2.7 cm (volume 13.8 cm^3^). **B** Given the single oligo-progression, the patient underwent percutaneous cryoablation with local curative intent; of note, the iceball (arrows) extending in the nearby soft-tissues, thus largely encompassing the target metastasis. **C** Axial CT image obtained at 3-month follow-up showing increased sizes (sized 9 cm; volume 265.3 cm^3^, corresponding to a volume increase significantly larger than 20%) of the target metastasis (arrows), thus being consistent with a progressive disease (PD)

To address this need, the BOREALIS Steering Committee developed criteria for assessing treatment response of BM following CA (Table 3). These criteria combine anatomical (lesion volume) and viability/metabolic features (contrast enhancement or radiotracer uptake) with clear quantitative thresholds for the standard response categories: complete response (CR), partial response (PR), stable disease (SD), and progressive disease (PD). The BOREALIS criteria were designed to remain applicable in real-world clinical settings, allowing sufficient flexibility to accommodate variability in imaging protocols across centres and the diversity of primary tumour origins. For instance, no restriction is imposed on the type of radiotracer used in PET-CT, as its selection should be guided by tumour histology and institutional practice. Examples of BM responses to CA are presented in Figs. 1, 2 and 3 and in Supplementary Figs. 1, 2.

**Table 3:** BOREALIS Tumour response criteria for bone metastases

	Size		CE-CT / CE-MRI		PET-CT
**CR**	Stable or decreased lesion volume	AND	Absence of contrast enhancement	or	Absence of radiotracer uptake
**PR**	Stable or decreased lesion volume	AND	≥ 30% decrease of viable lesion volume	or	≥ 25% reduction in SUV_max_
**SD**	*Any case that does not meet the criteria for CR, PR, PD*				
**PD**	Lesion volume increased ≥ 20%	OR	≥ 20% increase of viable lesion volume	or	> 25% increase in SUV_max_

CR: complete response, PR: partial response, SD: stable disease, PD: progressive disease, SUV: standardised uptake value. Assessment of tumour response shall be done per target lesion, ideally using the same imaging modality. CR and PR shall be assessed taking baseline as reference, PD shall be assessed taking as reference the best response since the first CA session

### Primary Outcome Measure

The primary outcome measure of BOREALIS is the **12-month local progression-free survival (LPFS) rate**, evaluated on a per-lesion basis by BCIR according to the BOREALIS response criteria.

### Secondary Outcome Measures

#### Secondary Effectiveness Outcome Measures

**Complete local tumour response** at 12 months, as well as **duration of response** (time from the first partial or complete response, until progression or death) will be assessed by BCIR on a per-lesion basis.

**Fracture-free survival** (time until fracture at treated site) and the **12-month fracture-free survival rate** will also be determined based on investigator-reported events and BCIR findings.

Additional effectiveness outcome measures, reported per patient, include **target BM progression-free survival** (time until progression of at least one target lesion), **bone-disease progression-free survival** (time until progression of any bone metastasis, including new metastases), **extraosseous disease progression-free survival** (time until progression outside the bone, including new metastases), **progression-free survival** (time until any progression, including new metastases, or death), **overall survival** (time until death) and **systemic therapy-free survival** (time until the next systemic anti-cancer treatment, excluding any adjuvant treatment that was part of the patient’s treatment plan.)

#### Feasibility

An essential component of BOREALIS is assessing the feasibility of a comprehensive treatment protocol that integrates within the same session i) biopsy (when indicated), ii) CA, and iii) bone consolidation by osteoplasty or osteosynthesis (when biomechanically indicated).

The study will additionally assess adherence to a comprehensive patient management framework comprising i) a pre-treatment visit, ii) regular clinical and radiological follow-ups (e.g., 1, 3, 6 and 12 months after treatment), and iii) multidisciplinary evaluation in the event of local tumour progression to consider further therapeutic strategies.

#### Safety

Safety will be assessed by monitoring the incidence and severity of adverse events graded according to the Common Terminology Criteria for Adverse Events (CTCAE) version 5.0 until the end of the study. These include, but are not limited to, infection, nerve injury, skin frostbite, non-target organ thermal injury, bleeding, symptomatic cement leakage, pain, nausea, vomiting, fever and sepsis.

Information on event severity (grades 1- 5), prolonged hospitalisation, need for additional intervention, and whether there was permanent patient impairment will be summarised as both the total number of events and the number and percentage of patients with at least one event. Data will be presented for all events, for unrelated or unlikely related events, and for events considered related to the CA procedure or device, or, when applicable, to the biopsy or bone consolidation, as determined by the PIs.

#### Patient-Reported Outcome Measures

Pain intensity and its impact on daily functioning will be assessed using the MD Anderson Cancer Centre Brief Pain Inventory – Short Form. This questionnaire employs numerical rating scales to measure pain severity through four items (worst, least, average, and current pain), as well as pain interference with seven domains of daily life (general activity, mood, walking ability, normal work, relations with others, sleep, and enjoyment of life). Single items assessing pain location and the use of pain-relieving treatments or medications are also included.

For each time point, the number and percentage of responses per item and dimension will be summarised for the categorical data, while the number of observations, mean, standard deviation, median, and range will be reported for the pain interference scales.

Patient-reported HRQoL will be evaluated using the EORTC QLQ-C30 questionnaire (version 3). This questionnaire includes 30 items covering five functional domains (physical, role, cognitive, emotional, and social functioning), three symptom domains (fatigue, nausea and vomiting, and pain), and a global health status/quality-of-life scale. Additional single items assess common cancer-related symptoms (e.g., dyspnoea, insomnia, appetite loss, constipation, diarrhoea). Data will be analysed according to the EORTC QLQ-C30 Scoring Manual (version 3, 2001) across all dimensions.

For both questionnaires, changes from baseline at each follow-up time point will be calculated per patient and the number of observations, mean, standard deviation, median, and range will be presented.

## Statistical Analysis

Descriptive statistics will be mainly used to report the outcome measures. The target of 42–50 participants was not determined through a formal sample size calculation but was assessed by the Steering Committee as a sufficiently large sample to provide meaningful information on the treatment’s effectiveness while remaining achievable within the planned enrolment period. To understand the level of uncertainty of the collected estimate, a method involving simulation and bootstrap resampling was employed, assuming a 1-year LPFS rate of 80%. With a sample size of 50 participants, a 95% confidence interval (CI) of 68%–90% can be expected, corresponding to a 20% probability of observing a local tumour control rate below 75%.

All participants who were planned to receive cryoablation will be included in the intent-to-treat (ITT) population, regardless of whether they received the planned procedure. In contrast, the modified intent-to-treat (mITT) population will only include participants who received cryoablation.

The results of the primary outcome measure (12-month LPFS rate) and its 95% confidence interval will be derived using the Kaplan–Meier estimate, on a per-lesion basis. Lesions without documented progression will be censored at the last assessment at which their status was known, including cases where patients died without prior lesion progression. For time-to-event outcome measures, median time-to-event and 95% CI will be calculated using the Kaplan–Meier estimator. The impact of different covariates on LPFS and all other time-to-event outcome measures will be assessed using a stratified log-rank test and a univariable Cox proportional hazards regression. Covariates with *p*-values < 0.1 and < 20% missing data will subsequently be included in a multivariable Cox proportional hazards regression model. Variables with *p*-values < 0.05 will be retained after backwards elimination, or if they contribute meaningfully to the model’s predictive performance. All other statistical tests will be based on a significance level of 5%, and estimates will be presented with 95% bilateral CIs. No adjustment for multiple testing will be done. For all time-to-event analyses, data will be censored at the last time point at which the patient’s status was assessed.

No imputation of missing data is planned for the analyses, and missing observations will be presented as percentage of the total.

Given the absence of planned confirmatory hypothesis testing, findings should be considered exploratory. Statistical analyses will be conducted using the latest available versions of R and RStudio.

## Discussion

Cryoablation has emerged as a very promising technique for managing BM, supported by a growing body of evidence demonstrating both its safety and substantial palliative benefit, and by several notable clinical and technical advantages (analgesic effect, multi-probe technique, visualisation of the iceball on cross-sectional imaging, etc.).

Current international guidelines endorse CA as a palliative treatment option for BM [14]. However, several retrospective studies suggest that beyond pain relief, CA may also achieve meaningful and durable local tumor control, with reported rates exceeding 80% at 16–35 months [8, 15], particularly in BM sized ≤ 2 cm [4, 7]. Although these retrospective results approach those achieved by stereotactic body radiation therapy (SBRT), which yields one-year control rates around 94% [16, 17], the quality and consistency of the available CA data remain insufficient to promote guideline inclusion for curative-intent treatment.

The absence of CA from oncologic guidelines has direct implications for patient access, despite several practical and oncologic advantages. Importantly, aside from high local tumor control rates, CA enables biopsy and bone consolidation to be performed during the same procedure [18], thereby offering immediate bone stabilization when indicated. This combined approach may significantly reduce the risk of secondary pathological fractures, which were reported in only 1.8% of cases (with an overall 2.3% major complication rate) in a large series of bone tumors undergoing CA [19]. These rates contrast sharply with the fracture risks associated with SBRT: up to 18% for vertebral sites [20] and up to 10% for extra-spinal ones [21, 22]. Moreover, while SBRT remains a highly effective option, its further limitations include decreased effectiveness in certain radio-resistant histologies, restrictions on repeat treatment due to cumulative dose constraints, and the logistical burden of multi-fraction regimens [23]. By contrast, CA is repeatable and compatible with concurrent systemic or local therapies, including radiation [24].

Despite these advantages, prospective real-world data evaluating the curative potential of CA remain scarce; and existing studies are limited by retrospective design, small cohorts, heterogeneous imaging and follow-up protocols, and lack of standardized response criteria. As a result, clinicians lack definitive evidence to guide patient selection, anticipate oncologic performance, or establish CA as a standard local treatment option for oligometastatic or oligoprogressive bone disease.

BOREALIS is designed to address these gaps. By focusing on patients undergoing CA with curative intent, BOREALIS aims to provide estimates of 12-month local progression-free survival rates, assessed using newly developed, ablation-specific tumor response criteria that integrate anatomical and functional imaging metrics.

## Conclusions

BOREALIS is designed to generate high-quality, prospective evidence to better define the role of CA in the contemporary management of BM requiring local control. By integrating clinical, procedural, radiological, and patient-reported outcomes, the study aims to inform future guideline development and clarify the position of CA among available therapeutic options for patients with oligometastatic or oligoprogressive bone disease.

## Supplementary Information

Below is the link to the electronic supplementary material.

Supplementary file1 (DOCX 6662 KB)
