# Supplementary material for: Curative-Intent Cryoablation for Bone Metastases: Study Protocol for BOREALIS, a Prospective Multicentre Observational Investigation
Source: Cardiovasc Intervent Radiol. 2026 Aug 19;49(10):2153–61. doi: 10.1007/s00270-026-04550-z (PMC13642440; doi:10.1007/s00270-026-04550-z)
Supplement: Supplementary file 1 [file 270_2026_4550_MOESM1_ESM.docx]

**Curative-intent cryoablation for bone metastases: Study protocol for BOREALIS, a prospective multicentre observational investigation - SUPPLEMENTARY FIGURES**

**Supplementary Figure 1. Example of complete response (CR) assessed by PET-CT.** 62-year-old female patient with a single biopsy-proven metastasis of breast cancer in the anterior aspect of T11 vertebral body, recurring after radiation therapy. (A) Axial Fluorodeoxyglucose (FDG) Positron Emission Tomography (PET-CT) image showing the FDG-avid (SUV_max_ 5) vertebral metastasis sized 0.9 cm (volume 0.2 cm^3^). (B) Given the oligo-metastatic scenario, the patient underwent percutaneous cryoablation with local curative intent, with two cryoprobes. (C) Axial FDG PET-CT obtained at 6-month follow-up showing absence of FDG uptake and size stability (0.9 cm; 0.2 cm^3^) of the target metastasis (arrow), thus being consistent with complete response (CR).

**Supplementary Figure 2. Example of complete response (CR) followed by progressive disease (PD) assessed by PET-CT.** 62-year-old male patient with a single biopsy-proven metastasis of renal cell carcinoma in the sacrum, recurring after radiation therapy. (A) Axial Fluorodeoxyglucose (FDG) Positron Emission Tomography (PET-CT) showing the recurring FDG-avid (SUV_max_ 5.2) metastasis (arrow), sized 1.0 cm (volume 0.9 cm^3^), close to the right S1 neuroforamen. Given the oligo-metastatic scenario, the patient underwent percutaneous cryoablation with local «curative intent». (B) Axial and (C) coronal computed tomography images showing cryoablation performed with two cryoprobes; of note the thermal protection achieved with a thermocouple and through perineural hydrodissection (arrows) of the right S1 nerve root. (D) Axial PET-CT image obtained at 3-month follow-up showing absence of FDG uptake and size stability (1.0 cm; volume 0.9 cm^3^; arrow) in the ablated zone, thus being consistent with a complete response (CR). (E) Axial PET-CT image obtained at 9-month follow-up showed substantial lesion stability in size (1.0 cm; volume 0.9 cm^3^) along with new FDG uptake (SUV_max_ 4.1, corresponding to a SUV_max_ increase higher than 25% compared to the previous PET-CT) in the ablation zone, thus being consistent a progressive disease (PD).
